# Dual Quantum Dot Molecular FRET Probes for Picomolar DNA Hexaplexing

**DOI:** 10.1002/smtd.70776

**Published:** 2026-06-22

**Authors:** Ruifang Su, Federico Pini, Nour Fayad, Kimihiro Susumu, Marta M. Natile, Igor L. Medintz, Thibault Gallavardin, Thomas J. Sørensen, Niko Hildebrandt

**Affiliations:** ^1^ Institut CARMeN UMR 6064 CNRS Université de Rouen Normandie INSA Rouen Normandie ENSICAEN Université de Caen Normandie Rouen France; ^2^ Nano‐Science Center & Department of Chemistry University of Copenhagen Copenhagen Denmark; ^3^ UMR 7021 CNRS, Laboratoire de Bioimagerie et Pathologies Universitè de Strasbourg Strasbourg France; ^4^ Consiglio Nazionale delle Ricerche (CNR) Istituto di Chimica della Materia Condensata e Tecnologie per l'Energia (ICMATE) Padova Italy; ^5^ Dipartimento di Scienze Chimiche Università di Padova Padova Italy; ^6^ CNRS UMR 5203, Inserm U1191, Université de Montpellier Institut de Génomique Fonctionnelle Montpellier France; ^7^ Optical Sciences Division, Code 5600 U.S. Naval Research Laboratory Washington, D.C. USA; ^8^ Center for Bio/Molecular Science and Engineering Code 6900 U.S. Naval Research Laboratory Washington, D.C. USA; ^9^ Department of Engineering Physics McMaster University Hamilton Ontario Canada

**Keywords:** biomarkers, biosensing, energy transfer, multiplexing, nanoparticles

## Abstract

Semiconductor quantum dots (QD) combine bright and color‐tunable photoluminescence (PL) with large surfaces. Thus, merging multiplexed PL detection and specific biorecognition on a single QD is in principle possible. By attaching multiple DNAs per QD and adjusting terbium (Tb) to QD Förster resonance energy transfer (FRET) with sub‐nanometer resolution, we designed dual‐color FRET‐nanoprobes with six distinguishable signals. The hexaplexing molecular probes combined two PL colors with three PL decays while requiring only a single excitation wavelength for rapid and simple biosensing. Implementation into a sensitive diagnostic assay was demonstrated via the specific quantification of six different DNA targets at picomolar concentrations (2 to 40 femtomole) from a single 200 µL sample. The orthogonality of specific biorecognition and FRET multiplexing on the nanosurface makes the QD‐based FRET probes extendable to the sensitive quantification of potentially any biomarker or biological interaction at even higher multiplexing capability. Our results demonstrate that luminescent nanoprobes are capable of translating sophisticated multiplexing approaches into relatively simple biosensing methods.

## Graphical abstract

Multiplexed quantification of six different DNA targets from a single 200 µL sample at picomolar concentrations was accomplished by spectrotemporal detection of time‐resolved FRET from a single Tb‐complex donor to two quantum dot acceptors using one‐color excitation and two‐color detection.

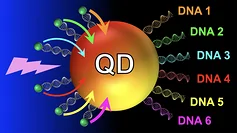

## 1 Introduction

With the continuous discovery of new disease biomarkers, biomolecular interactions, and biorecognition technologies, multiplexed optical biosensing of multiple targets from a single sample within a single experiment has become increasingly important for clinical and molecular diagnostics, biomolecular imaging, and environmental monitoring [1, 2, 3, 4, 5, 6, 7]. Photoluminescence (PL) detection is ideal for multiplexed biosensing due to its high sensitivity, rapid response, wide range of tunable fluorophores, and quantitative capabilities, allowing for the simultaneous detection of multiple targets with versatile signal readout, including PL intensities, spectra, and lifetimes [8, 9, 10, 11, 12, 13, 14].

The most frequently used strategy is based on spectral (or color) PL multiplexing with different fluorophores or luminescent nanomaterials [14, 15, 16, 17, 18, 19, 20]. To avoid multiple excitation wavelengths, PL probes with spectrally broad absorption, such as quantum dots (QDs) [21, 22, 23], or Förster resonance energy transfer (FRET) systems with one donor and multiple acceptors [15, 24, 25, 26, 27, 28], have been employed. QDs can also be applied for circumventing spectral crosstalk of multiple PL probes [29, 30], thereby improving the accuracy of multiplexed signals. Another approach is temporal PL multiplexing based on time‐resolved or time‐gated (TG) PL detection of lanthanide‐based FRET pairs [31, 32, 33]. Both spectral and temporal FRET multiplexing were also combined into spectrotemporal multiplexing using TG‐FRET detection of Tb‐dye FRET pairs at different wavelengths [34]. The FRET donor‐acceptor distance tuning that is necessary for accomplishing distinct PL lifetimes for temporal multiplexing was also demonstrated for lanthanide‐QD FRET pairs [35, 36]. However, the technology proof‐of‐concept has not been extended to actual multiplexed quantification of biomolecules, which is crucial for implementation into actual sensing applications.

Here, we demonstrate that spectrotemporal Tb‐to‐QD FRET can be applied to the quantification of multiple biological targets from a single low‐volume sample. By using a single Tb donor and two different QD acceptors and adjusting the Tb‐QD distance via specific peptide‐DNA scaffolds self‐assembled to the QD surfaces, we were able to design six distinguishable Tb‐QD FRET probes using a single excitation wavelength and dual color detection. The large surface of the QDs not only supplied the space for creating multiple Tb‐QD FRET pairs per QD and thereby bright and multiplexed PL probes, but it also provided additional space for attaching biological recognition elements for the detection of multiple specific targets. Although any polyhistidine‐tagged biomolecule can be self‐assembled to ZnS‐shelled QDs for biosensing [37, 38], we used DNA hybridization as a prototypical multiplexed bioassay. Aiming for multiplexed detection of very low target concentrations without signal or target amplification, and in order to apply a broadly used separation method, we used commercially available magnetic beads (MB) for improving assay sensitivity and reaching very low limits of detection (LOD) [39, 40, 41]. This approach enabled us to quantify six different DNA targets (four microRNA analogs and two mRNA‐derived housekeeping gene analogs) at low picomolar concentrations from a single 200 µL aqueous sample.

## 2 Results and Discussion

### 2.1 Design of the Hexaplexing Probes for DNA Hybridization Assays

**FIGURE 1:**
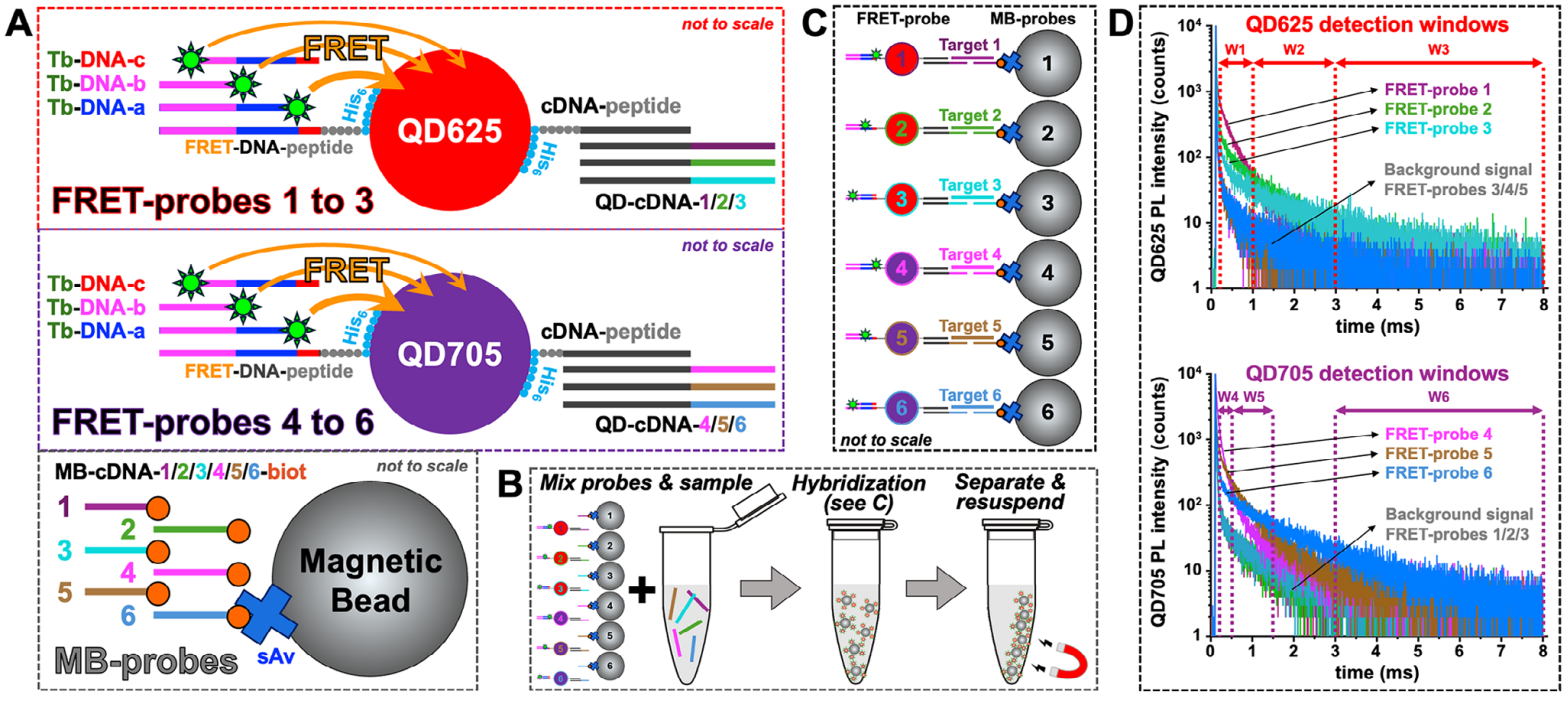
Hexaplexing of DNA targets based on Tb‐to‐QD FRET‐nanoprobes. (A) Schematics of the FRET‐probes (with QD625 and QD705 as central QDs) for multiplexing and MB‐probes (bottom) for separation. All FRET‐probes contained approximately 20 FRET‐DNA‐peptides and 10 cDNA‐peptides per QD (for simplicity, the schemes show only one of those DNA‐peptides per QD). Tb‐DNAs (for FRET) and QD‐cDNAs (for biorecognition) were hybridized to the DNA‐peptides on the QD surface. The configurations of the six FRET probes were 1: Tb‐DNA‐a/QD625/cDNA‐1; 2: Tb‐DNA‐b/QD625/cDNA‐2; 3: Tb‐DNA‐c/QD625/cDNA‐3; 4: Tb‐DNA‐a/QD705/cDNA‐4; 5: Tb‐DNA‐b/QD705/cDNA‐5; 6: Tb‐DNA‐c/QD705/cDNA‐6. Each MB‐probe (1 to 6) contained one type of cDNA (1 to 6) that was bound to the MB surface via biot‐sAv interaction. (B) Assay procedure: All probes were mixed with a target‐DNA‐containing sample, resulting in FRET‐probe‐target‐MB‐probe hybridization during 90 min incubation. Afterwards, the mixtures were washed three times via magnetic separation and resuspended for FRET measurements. (C) Schematic of the six different FRET‐probe‐target‐MB‐probe complexes. Note that the approximate diameters of QD625 (9.2 nm), QD705 (10.5 nm), and MB (1 µm) are very different, such that many FRET‐probes can be bound on a single MB‐probe. (D) The six different FRET‐probes (containing 3 FRET distances and 2 FRET acceptor colors) resulted in distinct FRET‐sensitized PL decay curves in the QD625 (top, PL at 607 ± 4 nm, excitation at 337.1 nm) and QD705 (bottom, PL at 716 ± 20 nm, excitation at 337.1 nm) detection channels. The distinct TG detection windows in the QD625 detection channel (W1: 0.2 to 1.0 ms; W2: 1.0 to 3.0 ms; W3: 3.0 to 8.0 ms) and the QD705 detection channel (W4: 0.2 to 0.5 ms; W5: 0.5 to 1.5 ms; W6: 3.0 to 8.0 ms) were selected for well‐balanced signal intensities.

FRET‐probes for target‐specific multiplexed PL detection and MB‐probes for target‐specific magnetic separation were prepared with distinct DNAs and DNA‐peptides (Figure 1A; all DNA and DNA‐peptide sequences are shown in Table 1). Six distinct FRET‐probes were assembled by attaching six target‐complementary DNAs (cDNAs) to the surfaces of two different QD acceptors, each of which was functionalized with a single type of Tb donor (Lumi4‐Tb) [42, 43] at three distinct distances via DNA hybridization.

**TABLE 1:** Sequences and modifications of DNA probes and targets.

Name	Sequence (5′‐3′)	Modification
FRET‐DNA‐peptide	TAGCTCGACAAAGTGCTCATAGTGCAGGTAG	5′‐H6SLGAAAGSGC‐SMCC‐amine
		
Tb‐DNA‐a	CTACCTGCACTATGAGCACTTTGTCGAGC	3′‐C3‐NH_2_
Tb‐DNA‐b	CTACCTGCACTATGAGC	3′‐C3‐NH_2_
Tb‐DNA‐c	CTATGAGCACTTTGTCGAGCTA	5′‐C3‐NH_2_
		
cDNA‐peptide	AGAACGGACTGAGCAGGAGAAAGATTTTCTATGGA	5′‐H6SLGAAAGSGC‐SMCC‐amine
		
QD‐cDNA1	TCTGATAAGCTATCCATAGAAAATCTTTCTCCTGCTCAGTCCGTTCT	
Target 1 (22 nt)	TAGCTTATCAGACTGATGTTGA	
MB‐cDNA1	AAAAAAAAAATCAACATCAG	5′‐biotin‐TEG
		
QD‐cDNA2	GATTAGCATTAATCCATAGAAAATCTTTCTCCTGCTCAGTCCGTTCT	
Target 2 (23 nt)	TTAATGCTAATCGTGATAGGGGT	
MB‐cDNA2	AAAAAAAAAAACCCCTATCAC	5′‐biotin‐TEG
		
QD‐cDNA3	CACAATCAAGACATTCTTTCCAGTCCATAGAAAATCTTTCTCCTGCTCAGTCCGTTCT	
Target 3 (46 nt)	CTGGAAAGAATGTCTTGATTGTGGAAGATATAATTGACACTGGCAA	
MB‐cDNA3	AAAAAAAAAATTGCCAGTGTCAATTATATCTTC	5′‐biotin‐C6
		
QD‐cDNA4	CTACTACCTCATCCATAGAAAATCTTTCTCCTGCTCAGTCCGTTCT	
Target 4 (22 nt)	TGAGGTAGTAGGTTGTATAGTT	
MB‐cDNA4	AAAAAAAAAAAACTATACAAC	5′‐biotin‐TEG
		
QD‐cDNA5	CCACTGTGAATCCATAGAAAATCTTTCTCCTGCTCAGTCCGTTCT	
Target 5 (21 nt)	TTCACAGTGGCTAAGTTCTGC	
MB‐cDNA5	AAAAAAAAAAGCAGAACTTAG	5′‐biotin‐TEG
		
QD‐cDNA6	ACCAGAGTTAAAAGCAGCCCTTCCATAGAAAATCTTTCTCCTGCTCAGTCCGTTCT	
Target 6 (42 nt)	AGGGCTGCTTTTAACTCTGGTAAAGTGGATATTGTTGCCATC	
MB‐cDNA6	AAAAAAAAAAGATGGCAACAATATCCACTTT	5′‐biotin‐C6

First, two types of hexahistidine (His_6_) tagged DNA‐peptides (FRET‐DNA‐peptide and cDNA‐peptide) were self‐assembled to the ZnS shells of QDs that were surface‐functionalized with compact ligands [44, 45, 46]. Tb‐labeled DNAs (Tb‐DNA‐a/b/c) were designed to hybridize to a specific fragment of the QD surface‐bound FRET‐DNA‐peptide to form Tb‐QD FRET pairs with distinct Tb‐QD distances (*R*) that were defined by the peptide‐linker plus 2 nucleotides (nt) for Tb‐DNA‐a, plus 14 nt for Tb‐DNA‐b, and plus 22 base pairs for Tb‐DNA‐c [36].

Second, six types of cDNA (QD‐cDNA‐1/2/3/4/5/6) complementary to one part of each DNA target sequence were hybridized to the cDNA‐peptide on each of the six Tb‐QD assemblies to create six distinct FRET probes (Figure 1A).

Finally, streptavidin (sAv) coated MBs were labeled with biotinylated (biot) cDNAs (MB‐cDNA‐1/2/3/4/5/6‐biot) that were complementary to another part of each DNA target sequence. Mixing of the FRET‐probes and MB‐probes with target‐DNA‐containing samples resulted in specific probe‐target hybridization. After magnetic separation from unbound probes and the sample matrix and resuspension (Figure 1B), the six FRET‐probes were used for quantifying the six distinct target DNAs (Figure 1C) in a background‐free solution.

Owing to the strong distance dependence (∼*R* ^−6^) of the FRET efficiency, the three different Tb‐to‐QD distances within the FRET‐probes resulted in distinct PL decays of the FRET‐quenched donors and the FRET‐sensitized acceptors (Figure 1D) [36, 47]. A combination of these three PL decay times with the two different acceptor QD colors (QD625 emitting around 625 nm and QD705 emitting around 705 nm) was used for higher‐order spectrotemporal hexaplexing (3 × 2 = 6) via six distinct TG PL detection windows (W1/2/3/4/5/6 in Figure 1D). Whereas the TG PL intensities in W1, W2, and W3 were mainly dependent on the concentrations or targets 1 to 3 (because targets 4 to 6 only led to background signal in the QD625 detection channel), the PL intensities in W4, W5, and W6 were mainly dependent on the concentrations or targets 4 to 6 (because targets 1 to 3 only led to background signal in the QD705 detection channel).

### 2.2 Photophysical Properties of the FRET‐Probes

**FIGURE 2:**
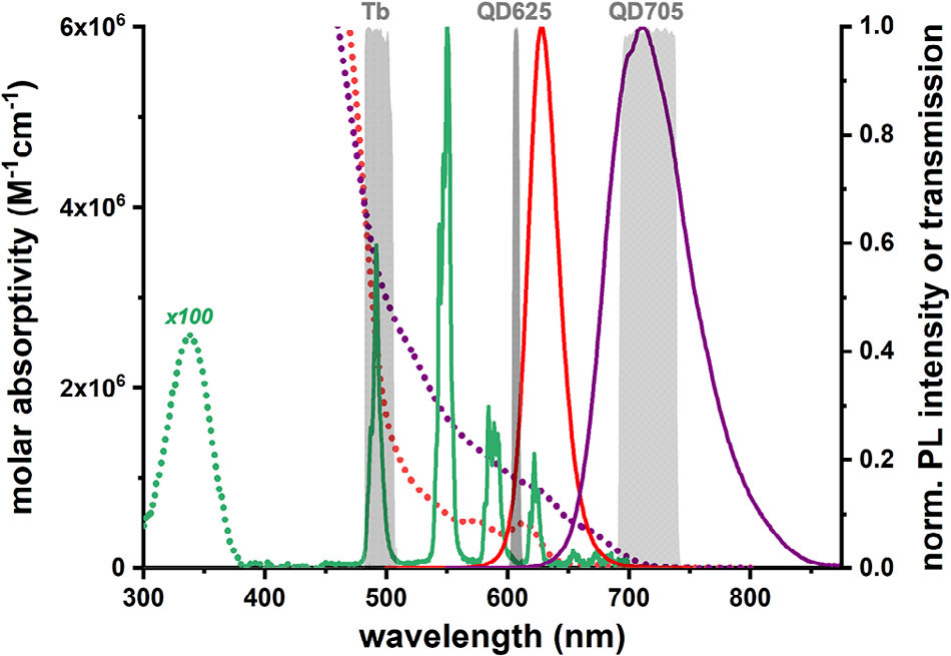
PL emission (solid lines) and absorption (dotted lines) spectra of Tb (green), QD625 (red), and QD705 (purple), and the filter transmission spectra (gray in the background) used for time‐resolved PL detection of Tb (494 ± 10 nm), QD625 (607 ± 4 nm), and QD705 (716 ± 20 nm).

The six FRET‐probes were comprised of one Tb donor and two different QD acceptors (QD625 and QD705), whose absorption and emission spectra are shown in Figure 2. In the case of a sufficiently close Tb‐QD distance, excitation of the Tb donor (around 340 nm) can result in FRET to either the QD625 or the QD705 acceptor because both QD absorption spectra overlap with the Tb emission spectrum. The PL spectra of Tb, QD625, and QD705 are well separated, such that each of the fluorophores can be detected independently, for which we used bandpass transmission filters (Figure 2). The QD625 filter transmission is particularly narrow because it needs to fit between two emission peaks of Tb in order to avoid spectral crosstalk of Tb emission into the QD625 detection channel. Considering that the QD625 is significantly brighter than a similar 605 nm emitting QD (both “QDots” from Thermo Fisher Scientific), measuring QD625 PL on the edge of its emission peak still provides high PL intensity. The two different QD PL signals (upon Tb excitation) provided the two spectral components of the multiplexed FRET‐probes.

The temporal multiplexing components of the FRET‐probes were accomplished by placing Tb at different distances to the QD surface, leading to different FRET efficiencies (*E*_FRET_). The spectral overlap of normalized Tb emission (${\bar{I}}_{D}(\lambda$)) and QD absorption (ε_*A*_(λ)) was used to calculate the FRET overlap integral ($J=\int {\bar{I}}_{D}\left(\lambda \right)\varepsilon_{A}\left(\lambda \right)\lambda^{4}d\lambda$). With *J*, an orientation factor κ^2^ = 2/3 for the dynamic averaging regime, a refractive index of *n* = 1.35 for aqueous buffers, and a Tb‐centered PL quantum yield of Φ_*D*_ = 0.80 ± 0.05, we estimated Förster distances (*R*_0_ =  0.021(κ^2^Φ_*D*_ *n* ^−4^ *J*)^1/6^) of 10.1 ± 1.5 nm for Tb‐QD625 and 11.4 ± 1.8 nm for Tb‐QD705 [47]. These *R*_0_ values represent the Tb‐QD distances at which FRET is 50% efficient. In addition, they are necessary to determine the actual Tb‐QD distances when using time‐resolved PL analysis (Equation 1) and the resulting PL lifetimes of the Tb donor in the absence of QD acceptors (τ_D_), of the Tb donor in the presence of QD acceptors, i.e., the FRET‐quenched Tb PL lifetime (τ_DA_), and of the QD acceptors in the presence of the Tb donor, i.e., the FRET‐sensitized QD lifetime (τ_AD_). For Tb‐QD acceptor pairs, the intrinsic PL lifetime of Tb is several orders of magnitude longer than the intrinsic PL lifetime of the QD and thus, τ_DA_ = τ_AD_ [47].

(1) $$\begin{array}{ccc} E_{\mathrm{FRET}} & = & \frac{{R_{0}}^{6}}{{R_{0}}^{6}+R^{6}}\;=\;1-\;\frac{\tau_{\mathrm{DA}}}{\tau_{D}}=\;1-\frac{\tau_{\mathrm{AD}}}{\tau_{D}}\;\Rightarrow R\;\\ & = & R_{0}\;{\left(\frac{\tau_{\mathrm{DA}}}{\tau_{D}-\tau_{\mathrm{DA}}}\right)}^{1/6}=R_{0}{\left(\frac{\tau_{\mathrm{AD}}}{\tau_{D}-\tau_{\mathrm{AD}}}\right)}^{1/6} \end{array}$$

We previously investigated Tb‐to‐QD surface distances using the DNA‐peptide surface attachment (cf. Figure 1A) and found that: i) one nt (in a single‐stranded DNA configuration after the peptide as valid for FRET‐probes 1, 2, 4, and 5) extends the Tb‐QD distance by ∼0.15 nm beyond the peptide part, thus ∼0.3 nm for 2 nt (FRET‐probes 1 and 4) and ∼2.1 nm for 14 nt (FRET‐probes 2 and 5); and ii) one base pair (in a double‐stranded DNA configuration after the peptide as valid for FRET‐probes 3 and 6) extends the Tb‐QD distance by ∼0.31 nm beyond the peptide part, thus ∼6.4 nm for 22 base pairs (FRET‐probes 3 and 6) [36]. Tb‐QD distances in FRET can be estimated as center‐to‐center distances, which means that the radius of the QD and the peptide extension (on the surface of the QD) must also be taken into account. For the Tb‐QD625 FRET system (using the same peptide and crosslinker on the DNA as in the present study), we previously found that the QD radius (∼4.6 nm) was extended by ∼2.9 nm via the peptide (+crosslinker to the DNA) to a total of approximately 7.5 nm (Tb‐QD center‐center distance) [36].

Considering a QD625 radius of ∼4.6 nm and a QD705 nm radius of ∼5.2 nm, we would have expected Tb‐QD distances of approximately 7.8 nm for FRET‐probe 1 (4.6 nm + 2.9 nm + 0.3 nm), 9.6 nm for FRET‐probe 2 (4.6 nm + 2.9 nm + 2.1 nm), 13.7 nm for FRET‐probe 3 (4.6 nm + 2.9 nm + 6.2 nm), 8.4 nm for FRET‐probe 4 (5.2 nm + 2.9 nm + 0.3 nm), 10.2 nm for FRET‐probe 5 (5.2 nm + 2.9 nm + 2.1 nm), 14.3 nm for FRET‐probe 6 (5.2 nm + 2.9 nm + 6.2 nm).

**FIGURE 3:**
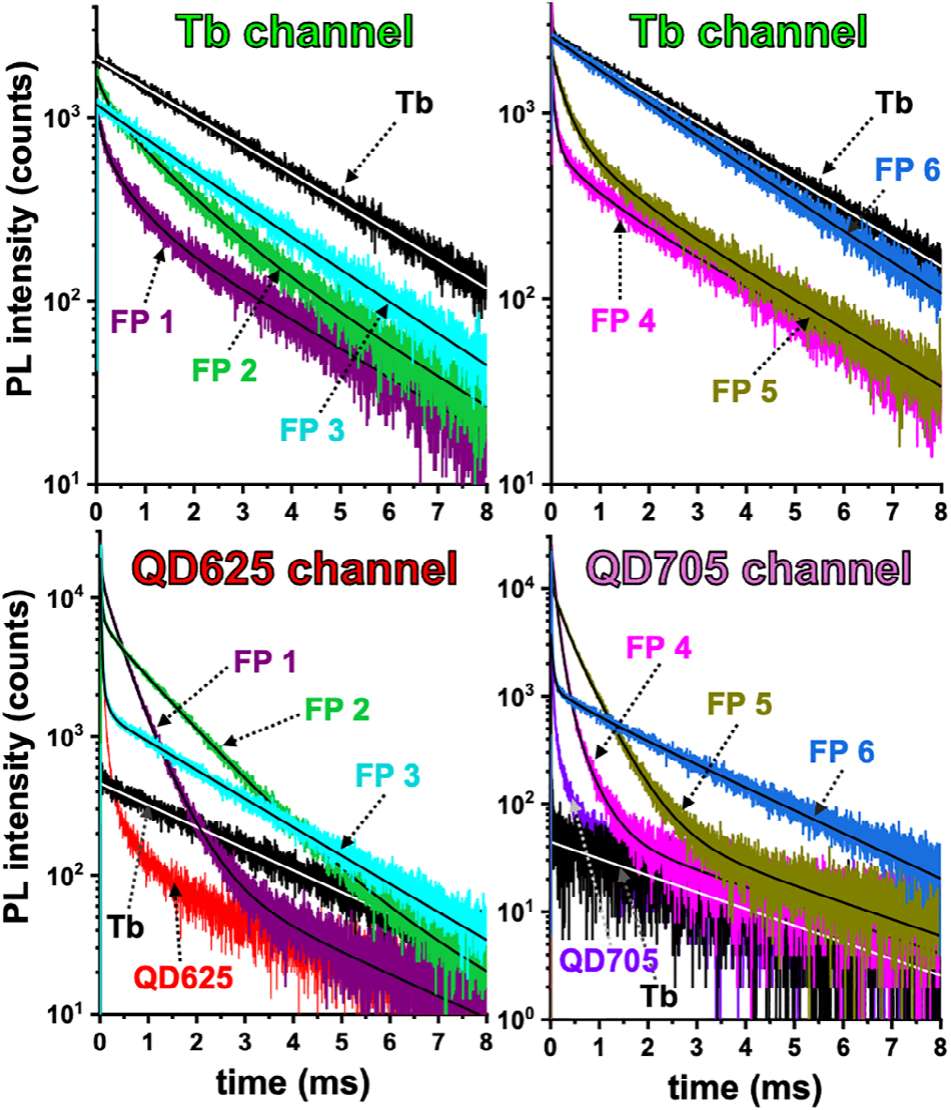
PL decay curves of the Tb donor in the absence of QD acceptors (black curves) and different FRET‐probes (FP—colored curves) in the Tb (top) and QD (bottom) detection channels (Tb: 494 ± 10 nm; QD625: 607 ± 4 nm; QD705: 716 ± 20 nm). Background PL signals of QD acceptors (QD625 and QD705 – red and purple curves) in the absence of Tb donors are also shown in the QD channels. QD PL signals do not crosstalk into the other channels. Tb PL signals are present in all channels, with higher crosstalk into the QD625 channel than into the QD705 channel (i.e., more intense black curve in the QD625 channel). The lines inside the decay curves present mathematical fits (cf. Tables S1 and S2). All decay curves were recorded following 337.1 nm excitation with a pulsed nitrogen laser.

In our previous work, QD625 were functionalized with dihydrolipoic acid‐appended poly(ethylene glycol) that probably resulted in a more radial extension of the peptide from the QD [36]. Thus, the Tb‐QD distances are likely a bit overestimated for the QDs coated with small ligands that were used in this work. Variations in shape (not perfectly spherical and monodisperse QDs) and flexibility in the DNA‐peptide structure are also contributing errors (which can be estimated to ∼10%) to these values. Despite these possible variations, the estimated Tb‐QD distances can provide a good orientation of what to expect regarding FRET efficiencies. More importantly, for temporal multiplexing, only the distinct PL decays of the FRET‐probes are decisive and not the exact Tb‐QD distances. For both the QD625 and the QD705 FRET‐probes, the PL decays were clearly distinguishable both in the Tb and the QD detection channels (Figure 3). Although the Tb PL decay is mono‐exponential, all FRET‐probes result in multi‐exponential PL decays, caused by Tb‐QD distance distributions due to the above‐mentioned QD shape variations and DNA‐peptide flexibility. The different distances result in multiple PL decay times, including cases for which the Tb donors are not quenched at all (long lifetime component corresponding to Tb).

Although efficient distinction of the PL decay curves is most important, they can also be used for analyzing the Tb‐QD distances via FRET, as done in our previous study [36]. Thus, we performed lifetime analysis of both FRET‐quenched (in the Tb detection channel) and FRET‐sensitized (in the QD detection channels) PL (see Supporting Information for details). PL decay fitting of the FRET lifetimes (Tables S1 and S2) resulted in Tb‐QD distances of 7.4 ± 0.8 nm (Tb channel) or 7.6 ± 0.8 nm (QD625 channel) for FRET‐probe 1, 8.8 ± 0.9 nm (Tb channel) or 9.3 ± 1.0 nm (QD625 channel) for FRET‐probe 2, 12.3 ± 1.3 nm (Tb channel) or 11.9 ± 1.2 nm (QD625 channel) for FRET‐probe 3, 7.2 ± 0.8 nm (Tb channel) or 7.4 ± 0.8 nm (QD705 channel) for FRET‐probe 4, 8.4 ± 0.9 nm (Tb channel) or 8.6 ± 0.9 nm (QD705 channel) for FRET‐probe 5, and 13.5 ± 1.4 nm (Tb channel) or 13.3 ± 1.4 nm (QD705 channel) for FRET‐probe 6. These distances were in very good agreement with the estimated values (7.5 vs 7.8 nm, 9.1 vs 9.6 nm, 12.1 vs 13.7 nm, 7.3 vs 8.4 nm, 8.5 vs 10.2 nm, and 13.4 vs 14.3 nm), which were between 0.2 and 1.8 nm longer, as expected due to the thicker QD coating used in our previous study (vide supra). The fit results not only confirmed the findings of our previous study, namely that time‐resolved FRET can be used for Tb‐QD distance measurements with high spatial resolution, by using two different QDs, they also demonstrated that our distance estimations can be used for designing Tb‐QD TG‐FRET probes for temporal multiplexing.

### 2.3 FRET‐Probe Assays for Six Different DNA Targets

The main objectives of our study were the development of spectrotemporally multiplexed FRET nanoprobes and the demonstration of their applicability to specific quantification of multiple biological targets at very low concentrations from a single sample. The translation to multiplexed diagnosis of biomarkers from real clinical samples was out of the scope of our current study, but will certainly be very interesting for future investigations. We have previously demonstrated that our prototypical target sensing approaches can be translated into the quantification of various clinically relevant biomarkers and clinical diagnosis in patient samples [11, 26, 48, 49, 50]. In this process, careful development, extensive characterization, and realistic proofs‐of‐concept of the new sensing technology are extremely important first steps, whereas thoughtful translation into real‐world applications follows in detailed subsequent studies.

**FIGURE 4:**
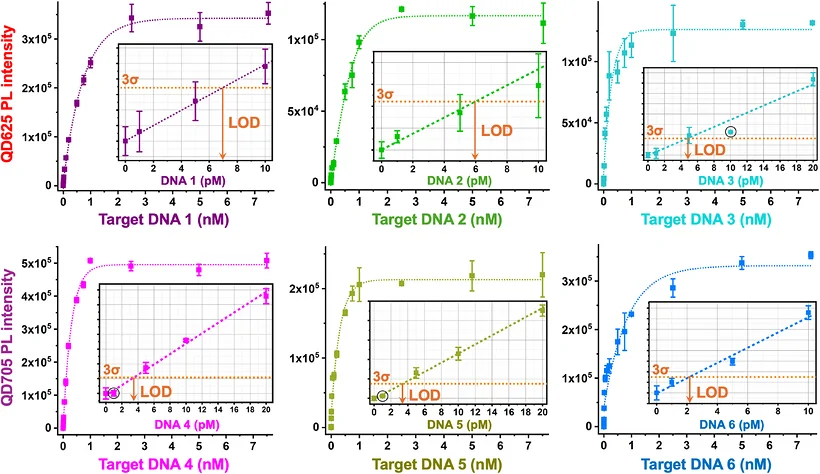
Single‐target hybridization assay calibration curves (FRET‐probes 1, 2, and 3 on top and FRET‐probes 4, 5, and 6 at the bottom from left to right) and determination of LODs (insets showing the low concentration range and linear fits through the shown data points) via three standard deviations (3σ) above the blank. A conservative estimation of σ was applied by calculating the average σ value of the four data points (circled data points for targets 3, 4, and 5 were not taken into account), shown in the insets. QD PL intensities were calculated by integrating the intensities in a detection window from 0.1 to 8.0 ms using the PL decay curves shown in Figure S1. Error bars present standard deviations from 10 measurements for blank samples (zero target concentration) and 3 measurements for all other samples. The shown target DNA concentrations are those in the reaction mixture (100 µL sample, 50 µL FRET‐probes, 50 µL MB‐probes).

Here, six different DNAs were used as prototypical targets to evaluate the analytical performance of the Tb‐QD FRET probes. Four of the targets were DNA analogs of the microRNA biomarkers miR‐21, miR‐155, let‐7a, and miR‐27, whereas two of the targets were DNA analogs of the mRNA‐derived housekeeping genes *HPRT1* (hypoxanthine phosphoribosyl transferase 1, nt 524–569) and GAPDH (glyceraldehyde 3‐phosphate dehydrogenase, nt 134–175). These targets did not only represent common biomarkers but also different sequence lengths (Target 1: 22 nt; Target 2: 23 nt; Target 3: 46 nt; Target 4: 22 nt; Target 5: 21 nt; and Target 6: 42 nt—cf. Table 1) for demonstrating the capability of quantifying nucleic acids of various sizes. Assay calibration curves (Figure 4) were recorded for all FRET‐probes. In these assays, 100 µL of sample containing the target DNA at different concentrations was mixed with 50 µL of the respective FRET‐probe at a concentration of 0.6 nm, and 50 µL of the respective MB‐probe at a concentration of 20 nm (MB concentration). The 200 µL mixtures were incubated for 90 min, followed by three washing steps via magnetic separation and resuspension in 150 µL of buffer for measurements on a time‐resolved fluorescence plate reader.

The calibration curves show the integrated PL intensities (from 0.1 to 8.0 ms after the excitation pulse—see Figure S1 for the PL decay curves recorded by the plate reader) of the FRET‐sensitized QDs over target concentration. The dynamic ranges of all probes span approximately three orders of magnitude (∼1 pM to ∼1 nm). Owing to the limited amount of QD‐cDNA (∼10 per QD, thus ∼1.5 nm in the 200 µL assay mixture), the PL intensities of the assay curves saturate at around 1.5 nm. LODs (three standard deviations above the blank—see insets in the graphs of Figure 4) were estimated as 7.0 ± 1.0 pM, 6.0 ± 0.9 pM, 5.0 ± 0.6 pM, 3.0 ± 0.3 pM, 3.0 ± 0.3 pM, and 2.0 ± 0.2 pM for FRET‐probes 1, 2, 3, 4, 5, and 6, respectively. The lower LODs for the QD705‐based FRET‐probes likely resulted from the lower Tb background PL in the QD705 detection channel (cf. Figure 2). The sub‐10 pM LODs and the broad dynamic PL intensity range demonstrate that all six FRET‐probes can be applied to distinguishing very low target concentrations, which is an important performance parameter for biomarker analysis.

### 2.4 Hexaplexing Assay

To realize the simultaneous quantification of very low concentrations of six different targets from a single low‐volume sample, we combined all FRET‐probes and MB‐probes. The assay procedure was similar to the one for single‐target detection. However, the different solutions (100 µL sample, 50 µL FRET‐probes, and 50 µL MB‐probes) contained all six target DNAs, all six FRET‐probes, and all six MB‐probes, and the six different TG detection windows W1 to W6 were applied to quantify all targets simultaneously. Because PL decays exponentially for each FRET‐probe and the PL intensities integrated over the different TG detection windows should be sufficiently high even for low target concentrations, the earlier windows (W1 and W4) were the narrowest and the later windows (W3 and W6) the broadest (cf. Figure 1D). Owing to the different PL decay kinetics (i.e., different PL lifetimes) of three probes in each QD detection channel (1 to 3 in the QD625 and 4 to 6 in the QD705 channel), they provided different PL intensity levels in the different TG detection windows. Since FRET‐probes 1 and 4 have the shortest (2 nt) Tb‐QD FRET‐distances (and thus high *E*_FRET_), they have the fastest decays (shortest  τ_AD_) and thus, their PL intensities mainly contribute to W1 and W4, respectively. Similar principles apply to the medium‐distance (i.e., medium *E*_FRET_ and medium  τ_AD_) FRET‐probes 2 and 5 that mainly contribute to W2 and W5, and the long‐distance (i.e., low *E*_FRET_ and long τ_AD_) FRET‐probes 3 and 6 that mainly contribute to W3 and W6.

Despite the distinct signals, all three FRET‐probes for each QD contribute to the three temporal windows (i.e., signal crosstalk into different channels) and the different contributions need to be separated (see “Mathematical treatment of hexaplexing” in the Materials and Methods section). Although the separation procedure is straightforward and, in principle, many more FRET distances with distinct PL decay curves and detection windows can be applied for higher levels of multiplexing, one needs to consider the PL intensity (the higher the intensity, the lower the LOD) and the specificity (the more distinct the decay curves, the better the probe distinction) in each channel. Thus, higher levels of temporal multiplexing usually have the drawback of lower sensitivity and selectivity.

**FIGURE 5:**
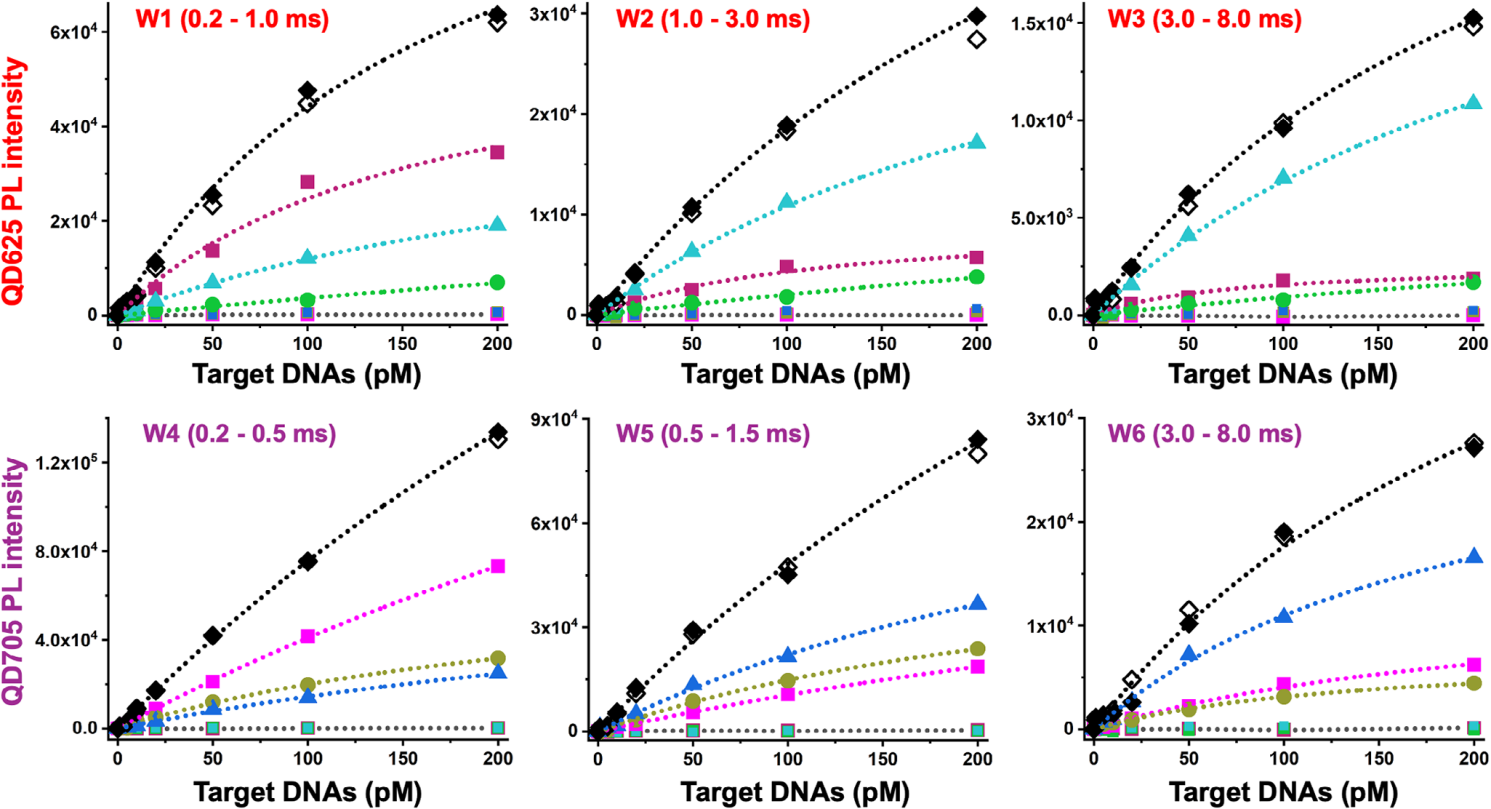
Assay calibration curves (TG PL intensities of FRET‐sensitized QD625 (top) and QD705 (bottom) as a function of DNA target concentration in the six detection windows W1 to W6) for individual targets (Target 1: purple squares; Target 2: green circles; Target 3: cyan triangles; Target 4: magenta squares; Target 5: dark yellow circles; Target 6: blue triangles) and all six targets (black diamonds). The sum of the PL intensities of all individual targets is also shown for comparison (open diamonds). Targets 1 to 3 do not contribute to FRET‐sensitization of QD705, and targets 4 to 6 do not contribute to FRET‐sensitization of QD625. Therefore, each graph shows concentration‐dependent PL intensities for only three targets (whereas the PL intensities for the other three targets remain at background levels). Curves are fits of the data points using Equation (2). QD PL intensities were calculated by integrating the intensities in the corresponding detection windows (W1 to W6) using the PL decay curves shown in Figure S2.

Considering that very low target concentrations are most relevant for early diagnostics and that the LODs for single‐target detection were between circa 2 and 7 pM, we focused on quantifying the six DNA targets in a concentration range between 10 and 200 pM. Assay calibration curves were acquired for each individual target as well as all six targets in each detection window (Figure 5). Because the spectral detection channels (QD625 and QD705 bandpass filters—cf. Figure 2) do not result in spectral crosstalk, FRET‐probes 1, 2, and 3 create only QD625 PL signals, whereas FRET‐probes 4, 5, and 6 create only QD705 PL signals.

Target hexaplexing from a single sample was solved via triplexing in the two (QD625 and QD705) detection channels (3 × 2 = 6). Quantification of the six DNA targets from a single mixed sample was accomplished by fitting the calibration curves with a simple Michaelis‐Menten equation and solving the concentrations of the different target DNAs via a straightforward root‐finding algorithm (for details, see Materials and Methods section, including Equations (2), (3), (4), (5), (6), (7), (8), (9), (10), (11), (12), (13)).

**FIGURE 6:**
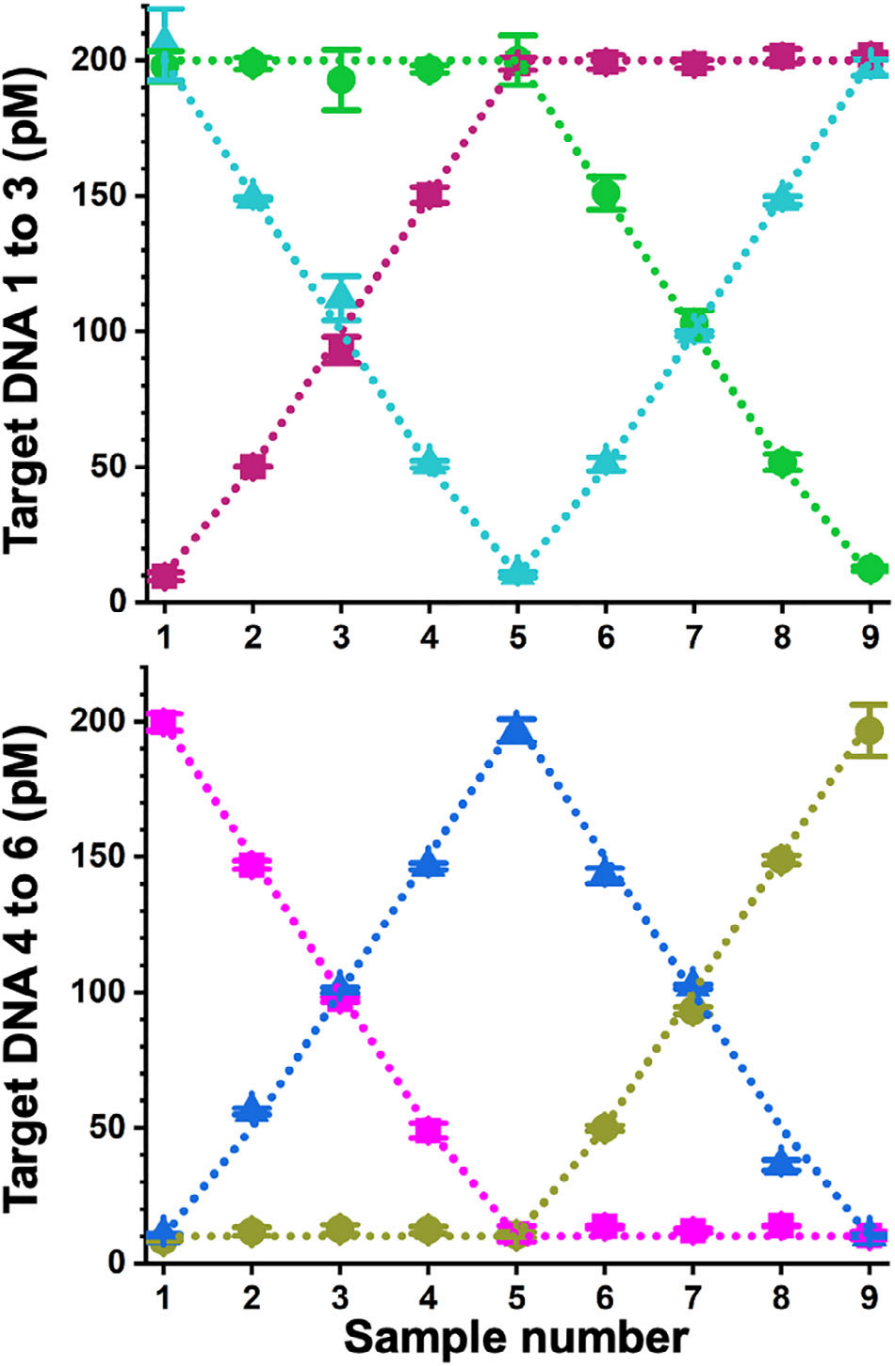
Recovery of varying concentrations of target DNAs 1 to 6 from nine different samples using spectro‐temporal hexaplexing FRET assays. For better visibility of the different data points and because targets 1 to 3 were quantified via FRET‐sensitized QD625 emission and targets 4 to 6 were quantified via FRET‐sensitized QD705 emission, the results are separated into two graphs. Dotted lines represent known concentrations, whereas data points represent FRET‐assay results. Error bars are from triplicate measurements of each sample. The PL decay curves representing the experimental data used for analyzing the different time windows are shown in Figure S3.

To test the hexaplexing method, we prepared nine samples containing all six target DNAs at concentrations between 10 and 200 pM, corresponding to 2 to 40 fmol of DNA in the 200 µL samples. Hexaplexed FRET recovery of the six DNA targets in the nine different samples was both accurate and precise, with only small deviations from the actual concentrations (Figure 6). These results demonstrated simple, rapid, and reliable higher‐order multiplexed target quantification at very low (picomolar) concentrations in low‐volume samples (200 µL) with a single excitation wavelength and only two emission wavelengths.

## 3 Conclusions

The quantification of multiple biomarkers from a single low‐volume sample is of high interest for biological, biochemical, clinical, and environmental research and sensing applications. For example, multiplexing is commonly used in DNA assays for viral target screening or in RNA assays for biomarker‐based disease diagnostics. Higher‐order multiplexing, which combines different multiplexing strategies, has a large potential for simultaneously quantifying many targets because the multiplexing digits of the different strategies multiply. However, the multiple parameters that need to be adapted for such higher‐order multiplexing approaches make implementation into simple and quantitative assays extremely challenging. Here, we combined dual color multiplexing with triple lifetime multiplexing into higher‐order hexaplexing. FRET provided both the color and the time codes of our hexaplexing nanoprobes. Excitation of a common Tb donor (one excitation wavelength for all probes) and FRET to two different QD acceptors and at three different Tb‐QD distances resulted in three distinct PL decays per each QD detection channel. i.e., six distinguishable PL decay curves. Simple and rapid TG PL intensity quantification in six distinct temporal detection windows was used to specifically separate the signals of the six Tb‐QD FRET‐probes. The large surface‐to‐volume ratios of the QDs provided two main advantages: i) Several Tb‐donors could be attached per QD, leading to bright probes; ii) There was sufficient space to also attach biorecognition molecules, such that various binding events per probe were possible.

We applied DNA hybridization assays as a prototypical sensing approach. Six different DNA targets at picomolar concentrations (femtomole amounts) were captured from a single 200 µL sample via specific sandwich hybridization between the six different FRET‐probes and MBs for efficient separation from unbound probes and the sample matrix. Rapid detection using a fluorescence plate reader allowed for the specific recovery of all six targets in the 10 to 200 pM concentration range from different sample mixtures. This sensitive DNA hexaplexing demonstrated that higher‐order multiplexed FRET nanoprobes can be applied to the actual quantification of free biomarkers in aqueous media, which presents a significant advance toward translation into molecular diagnostics. Importantly, the multiplexed probes are independent of the binding event to be quantified, and it should be straightforward to implement them into other assays, biosensors, or imaging applications. Moreover, considering that we had previously distinguished TG‐FRET with up to five different QDs [30], a possible extension to pentadecamultiplexing using five QD probes and three different FRET‐distances (5 × 3 = 15) or even higher multipliers should be feasible.

Although LODs are difficult to compare for biosensors with different probes, targets, and measurement conditions, hybridization assays based on fluorescent nanoparticles and magnetic separation recently demonstrated sub‐femtomolar LODs [51]. Thus, further optimization of our FRET probes and assay conditions can potentially lead to the detection of even lower target concentrations than the 10 to 200 pM range demonstrated in our study. While temporal multiplexing can distinguish different targets via a single measurement from a single sample, the method also has its limitations. Higher levels of temporal multiplexing result in lower sensitivity and selectivity, including inefficient distinction of targets with high concentration ratios (ratio of highest to lowest concentration of different targets). Our proof‐of‐concept demonstrated a target concentration ratio of 20:1 (10 to 200 pM—cf. Figure 6) using three temporal detection channels, but extreme differences in target concentrations (1000:1 or more) may be difficult to accomplish in the temporal domain. The spectral (color) detection channels are largely independent from each other and thus, higher sensitivity, selectivity, and target concentration ratios are feasible via spectral multiplexing. It is important to keep such limitations in mind when designing multiplexed assays. Multiplexing always requires the consideration of the analytical targets, sample type and volume, and simplicity, time, cost, sensitivity, and selectivity of the assay and instrumentation. If, for example, sample volume and duration of the assay have no limits, the best multiplexing performance will be accomplished by separating the sample and performing one separate assay for each target.

Overall, our results show that thoughtful design of FRET‐nanoprobes, careful characterization of their photophysical properties, implementation of theoretical modeling, and representative proof‐of‐concept bioassays are capable of translating even sophisticated multiplexing approaches into simple and rapid detection methods for biosensing and molecular diagnostics.

## 4 Experimental Section

### 4.1 Materials

Lumi4‐Tb‐NHS (Tb) was provided by Lumiphore Inc. 625 nm emitting organic Qdots (QD625, core‐shell diameter of ∼9.2 nm) [36], 705 nm emitting organic Qdots (QD705, core‐shell diameter of ∼10.5 nm) [52] and Dynabeads MyOne Streptavidin C1 magnetic beads (MB, diameter of 1 µm) covalently coupled to streptavidin were purchased from Thermo Fisher Scientific. DNA‐peptides were purchased from Biosynthesis. All other oligonucleotides were purchased from Eurogentec. All sequences and modifications of nucleic acids used in this work are summarized in Table 1. Tris(hydroxymethyl)‐aminomethane, ethylenediaminetetraacetic acid (EDTA), hydrochloric acid (HCl), sodium hydroxide (NaOH), sodium tetraborate decahydrate (Na_2_B_4_O_7_·10H_2_O), sodium bicarbonate (NaHCO_3_), bovine serum albumin (BSA), and HEPES were purchased from Sigma–Aldrich. NaCl was purchased from Duchefa. Zeba Spin Desalting Columns (7 kDa MWCO) were purchased from Thermo Fisher Scientific. All chemicals were used as received. High‐quality Milli‐Q water with a resistivity of 18.2 MΩ cm was used for preparing solutions.

### 4.2 Tb‐DNA Conjugation

Three different Tb‐DNA conjugates were prepared by mixing Lumi4‐Tb‐NHS in concentration excess to three different amino‐functionalized oligonucleotides (Tb‐DNA‐a, b or c as shown in Table 1) in 100 mm carbonate buffer at pH 9.0, respectively. The mixtures were incubated overnight at 4 C and purified 3 times with HEPES buffer (100 mm, pH 7.4) by Zeba Spin Desalting Columns (7 kDa MWCO). Tb concentration of the conjugate was determined by measuring the absorbance at ∼340 nm using a molar absorptivity of ∼26 000 M^−1^ cm^−1^ as provided by the manufacturer. DNA was quantified by absorbance measurements at ∼260 nm. The conjugation ratio was determined to be higher than 0.8 Tb/DNA by a linear combination of the respective absorbance values of Lumi4‐Tb‐NHS and DNA within the conjugate.

### 4.3 Tb‐to‐QD FRET‐Probes

The Tb‐DNA conjugates were used to construct the different Tb‐QD FRET configurations with different Tb‐to‐QD distances. Specific Tb‐DNA conjugates were mixed with the FRET‐DNA‐peptide, QD625 or QD705, cDNA‐peptide, and the corresponding QD‐cDNA at a concentration ratio of 20: 20: 1: 10: 10 (c_Tb‐DNA_: c_FRET‐DNA‐peptide_: c_QD_: c_cDNA‐peptide_: c_QD‐cDNA_). The mixtures were prepared in hybridization buffer (20 mm Tris‐Cl, 500 mm NaCl, 0.1% BSA, pH 8.0), incubated at 37 °C for 30 min, then at room temperature for 3 h, and subsequently stored at 4 °C for long‐term use. During incubation, the His_6_ tags of FRET‐DNA‐peptide and cDNA‐peptide were attached to the Zn‐rich QD surface via metal affinity‐mediated self‐assembly, which allowed the Tb‐DNA to hybridize to FRET‐DNA‐peptide and form Tb‐QD‐cDNA FRET‐probes.

### 4.4 MB‐cDNA Conjugation

The conjugates of biotin‐cDNA with streptavidin MBs were prepared according to the protocol from Thermo Fisher Scientific [53]. First, MBs were washed according to the following steps: (i) Resuspend the beads in the vial (i.e., vortex for >30 s or tilt and rotate for 5 min); (ii) Transfer the desired volume of beads to a new tube; (iii) Add an equal volume (or at least 1 mL) of washing buffer (5 mm Tris‐HCl, 0.5 mM EDTA, and 1 m NaCl, pH 7.5) and resuspend; (iv) Place the tube on a magnet for 1 min and discard the supernatant; (v) Remove the tube from the magnet and resuspend the washed beads in 1 mL washing buffer; (vi) Repeat steps iv–v twice, for a total of three washes. Second, six different MB‐cDNAs (MB‐cDNA1 to 6) were immobilized onto MBs through biot‐sAv non‐covalent binding according to the following steps: (i) Resuspend washed MBs in 2× washing buffer (10 mM Tris‐HCl, 1 mM EDTA, and 2 m NaCl, pH 7.5) to a final concentration of 5 µg/µL (half of the original concentration (10 mg/mL)); (ii) Add an equal volume of biotinylated DNA (the binding ratio between DNA and MBs is 400 pmol cDNA per mg of MB). Optimal binding occurs when the NaCl concentration is reduced from 2 to 1 m; (iii) Incubate for 15 min at room temperature using gentle rotation; (iv) Separate the DNA‐coated MBs with a magnet for 2–3 min; (v) Wash the coated beads 2–3 times with 1× washing buffer; (vi) Resuspend the beads with the immobilized cDNAs in hybridization buffer (20 mm Tris‐Cl, 500 mm NaCl, 0.1% BSA, pH 8.0) for downstream applications.

### 4.5 Spectroscopic Characterization

Absorption spectra (Cary 60 UV–vis Spectrophotometer, Agilent) and emission spectra (Fluorolog‐3, HORIBA) for Tb and QD samples were recorded in HEPES (100 mm, pH 7.4) and Na‐tetraborate buffer (100 mm Na‐tetraborate, 0.1 m NaCl, pH 8.5), respectively.

### 4.6 Single and Hexaplexed DNA Target Sensing

All FRET assays were prepared in hybridization buffer (20 mm Tris‐Cl, 500 mm NaCl, 0.1% BSA, pH 8.0). For single‐target sensing assays, 50 µL (0.6 nm, concentration determined by QDs) of the corresponding FRET‐probes, and 50 µL (20 nm) of the corresponding MB‐cDNAs were mixed with 100 µL specific DNA target (at different concentrations). For hexaplexing assays, 50 µL of a mixed solution containing six types of FRET‐probes 1 to 6 (each at 0.6 nm) and 50 µL of a mixed solution containing six types of MB‐probes 1 to 6 (each at 20 nm) were combined with 100 µL of a mixed solution containing six types of DNA targets at varying concentrations. All samples were prepared in triplicate, except for the blank samples (target concentration = 0), which were prepared ten times. The mixtures were incubated at 37°C for 30 min, then followed by cooling down to room temperature (20°C) for 60 min. After incubation, the mixtures were washed three times with hybridization buffer (20 mM Tris‐Cl, 500 mm NaCl, 0.1% BSA, pH 8.0) through magnetic separation according to the procedure mentioned above and then resuspended with 150 µL hybridization buffer (20 mm Tris‐Cl, 500 mm NaCl, 0.1% BSA, pH 8.0). 140 µL were taken from the resuspended solution (150 µL) and measured in black 96‐well microtiter plates on a fluorescence lifetime plate reader (Edinburgh Instruments) using 4000 detection bins of 2 µs integration time and a pulsed nitrogen laser (MNL 100, LTB Berlin) for excitation (337.1 nm, 20 Hz). The PL intensities of Tb donor, QD625, and QD705 acceptor were measured by using optical bandpass filters with 494/20 nm (Semrock) for the donor channel and 607/8 nm (Delta) and 716/40 nm (Semrock) for the acceptor channels.

### 4.7 Mathematical Treatment of Hexaplexing

All calibration curves could be very well fit using a simple Michaelis‐Menten model (Equation 2):

(2) $$f_{Wi}\left(c_{Tj}\right)=\frac{I\left(Wi,Tj\right)\cdot c_{Tj}}{K\left(Wi,Tj\right)+c_{Tj}}$$

Here, *I*(Wi,Tj) is the maximum (i.e., saturation) TG PL intensity in each detection window (*W*i, with i = 1 to 6) for each target (*T*j, with j = 1 to 6), *c_Tj_* is the concentration of each target, and *K*(*W*i,*T*j) is a constant that is equal to the concentration at which the TG PL intensity is half of *I*(*W*i,*T*j). We used this equation because it showed an excellent fit to the experimental data, and not with the aim of modeling a biochemical reaction (the Michaelis‐Menten model is usually used for enzyme kinetics). Fitting of the single‐target calibration curves with Equation (2) resulted in the two parameters ***I*** and ***K*** for each target in each detection window, and the 3 × 3 matrices for each (QD625 and QD705) detection channel (Equations 3 and 4) can be created:

(3) $$\begin{array}{ccc} & & I_{QD625}=\left[\begin{array}{ccc} I\left(W1,T1\right) & I\left(W1,T2\right) & I\left(W1,T3\right)\\ I\left(W2,T1\right) & I\left(W2,T2\right) & I\left(W2,T3\right)\\ I\left(W3,T1\right) & I\left(W3,T2\right) & I\left(W3,T3\right) \end{array}\right];\\ & & I_{\mathrm{QD}705}=\left[\begin{array}{ccc} I\left(W4,T4\right) & I\left(W4,T5\right) & I\left(W4,T6\right)\\ I\left(W5,T4\right) & I\left(W5,T5\right) & I\left(W5,T6\right)\\ I\left(W6,T4\right) & I\left(W6,T5\right) & I\left(W6,T6\right) \end{array}\right]\; \end{array}$$

(4) $$\begin{array}{ccc} & & K_{\mathrm{QD}625}=\left[\begin{array}{ccc} K\left(W1,T1\right) & K\left(W1,T2\right) & K\left(W1,T3\right)\\ K\left(W2,T1\right) & K\left(W2,T2\right) & K\left(W2,T3\right)\\ K\left(W3,T1\right) & K\left(W3,T2\right) & K\left(W3,T3\right) \end{array}\right];\\ & & \;K_{\mathrm{QD}705}=\left[\begin{array}{ccc} K\left(W4,T4\right) & K\left(W4,T5\right) & K\left(W4,T6\right)\\ K\left(W5,T4\right) & K\left(W5,T5\right) & K\left(W5,T6\right)\\ K\left(W6,T4\right) & K\left(W6,T5\right) & K\left(W6,T6\right) \end{array}\right]\; \end{array}$$

The experimental data collected the different time windows in the two spectral detection channels can be described as vectors *F*_QD625_ and *F*_QD705_ (Equation 5):

(5) $$F_{\mathrm{QD}625}=\left[\begin{array}{ccc} f_{W1} & f_{W2} & f_{W3} \end{array}\right];\;F_{\mathrm{QD}705}=\left[\begin{array}{ccc} f_{W4} & f_{W5} & f_{W6} \end{array}\right]\;$$

Due to the linear relationship between single sensing and triplexing (the sum of the single signals equals the signal of the sum of targets—cf. Figure 5), the signal in each channel (the vectors with experimental data) can be described as a function of the target concentrations (Equations 6 and 7):

(6) $$\begin{array}{ccc} \mathrm{For}\;W1\;\mathrm{to}\;W3\;(i.e.,\;\mathrm{QD}625) & & :f_{Wi}\;\left(c_{T1},\;c_{T2},c_{T3}\right)\\ & & =\sum_{j=1}^{3}\frac{I\left(Wi,Tj\right)\;\cdot \;c_{Tj}}{K\left(Wi,Tj\right)+c_{Tj}} \end{array}$$

(7) $$\begin{array}{ccc} \mathrm{For}\;W4\;\mathrm{to}\;W6\;(i.e.,\;\mathrm{QD}705) & & :f_{Wi}\;\left(c_{T4},\;c_{T5},c_{T6}\right)\\ & & =\sum_{j=4}^{6}\frac{I\left(Wi,Tj\right)\;\cdot \;c_{Tj}}{K\left(Wi,Tj\right)+c_{Tj}} \end{array}$$

The concentrations of multiple targets in a mixed sample can be solved via the Newton‐Raphson method that starts with an initial guess of a concentration (*C* ^(n = 0)^) and finds better solutions for each iteration *n*+1 (Equation 8):

(8) $$C^{\left(n+1\right)}=C^{\left(n\right)}\;-\frac{F\left(C^{\left(n\right)}\right)}{J\left(C^{\left(n\right)}\right)}$$

where *F* is the set of functions obtained for *C* ^(n)^ (Equations 9 and 10):

(9) $$\mathrm{For}\;W1\;\mathrm{to}\;W3\;(i.e.,\;\mathrm{QD}625):\;C^{\left(n\right)}=\left[\begin{array}{ccc} c_{T1}^{\left(n\right)} & c_{T2}^{\left(n\right)} & c_{T3}^{\left(n\right)} \end{array}\right]\;$$

(10) $$\mathrm{For}\;W4\;\mathrm{to}\;W6\;(i.e.,\;\mathrm{QD}705):\;C^{\left(n\right)}=\left[\begin{array}{ccc} c_{T4}^{\left(n\right)} & c_{T5}^{\left(n\right)} & c_{T6}^{\left(n\right)} \end{array}\right]\;$$

and *J* is the Jacobian for *C* ^(*n*)^ (Equations 11 and 12):

(11) $$\mathrm{For}\;W1\;\mathrm{to}\;W3\;(i.e.,\;\mathrm{QD}625):J=\left[\begin{array}{ccc} \frac{\partial f_{W1}}{\partial c_{T1}} & \frac{\partial f_{W1}}{\partial c_{T2}} & \frac{\partial f_{W1}}{\partial c_{T3}}\\ \frac{\partial f_{W2}}{\partial c_{T1}} & \frac{\partial f_{W2}}{\partial c_{T2}} & \frac{\partial f_{W2}}{\partial c_{T3}}\\ \frac{\partial f_{W3}}{\partial c_{T1}} & \frac{\partial f_{W3}}{\partial c_{T2}} & \frac{\partial f_{W3}}{\partial c_{T3}} \end{array}\right]\;$$

(12) $$\mathrm{For}\;W4\;\mathrm{to}\;W6\;(i.e.,\;\mathrm{QD}705):J=\left[\begin{array}{ccc} \frac{\partial f_{W4}}{\partial c_{T4}} & \frac{\partial f_{W4}}{\partial c_{T5}} & \frac{\partial f_{W4}}{\partial c_{T6}}\\ \frac{\partial f_{W5}}{\partial c_{T4}} & \frac{\partial f_{W5}}{\partial c_{T5}} & \frac{\partial f_{W5}}{\partial c_{T6}}\\ \frac{\partial f_{W6}}{\partial c_{T4}} & \frac{\partial f_{W6}}{\partial c_{T5}} & \frac{\partial f_{W6}}{\partial c_{T6}} \end{array}\right]\;$$

with the following elements (Equation 13):

(13) $$\frac{\partial f_{Wi}}{\partial c_{Tj}}=\frac{I\left(Wi,Tj\right)\cdot K\left(Wi,Tj\right)}{{\left(K\left(Wi,Tj\right)+c_{Tj}\right)}^{2}}\;$$

Using the experimental data from the different time windows (*f_W1_* to *f_W6_*) and providing a starting concentration *C* ^(0)^, the Newton‐Raphson method was used to fit the concentrations of Target 1 to Target 6 until the experimental data were in agreement with the fit (i.e., $f_{Wi}^{\exp.}-f_{Wi}\;\left(c_{T1/T4},c_{T2/T5},c_{T3/T6}\right)=0$).

## Conflicts of Interest

The authors declare no conflicts of interest.

## Supporting information




**Supporting File**: smtd70776‐sup‐0001‐SuppMat.docx.

## Data Availability

The data that support the findings of this study are available in the supplementary material of this article.
