# Supplementary material for: Dual Quantum Dot Molecular FRET Probes for Picomolar DNA Hexaplexing
Source: Small Methods. 2026 Jun 22;10(16):e70776. doi: 10.1002/smtd.70776 (PMC13505750; doi:10.1002/smtd.70776)
Supplement: Supplementary file 1 [file SMTD-10-e70776-s001.docx]

**----SUPPORTING INFORMATION----**

**Dual Quantum Dot Molecular FRET Probes for Picomolar DNA Hexaplexing**

Ruifang Su,^1,2,3^ Federico Pini,^1,4,5^ Nour Fayad,^1,6^ Kimihiro Susumu,^7^ Marta M. Natile,^4,5^ Igor L. Medintz,^8^ Thibault Gallavardin,^1^ Thomas J. Sørensen,*^2^ and Niko Hildebrandt*^9^

*^1^ Institut CARMeN UMR 6064, Univ Rouen Normandie, CNRS, INSA Rouen Normandie, Univ Caen Normandie, ENSICAEN, F-76000 Rouen, France.*

*^2^ Nano-Science Center & Department of Chemistry, University of Copenhagen, 2100 Copenhagen, Denmark.*

*^3^ Laboratoire de Bioimagerie et Pathologies, UMR 7021 CNRS, Universitè de Strasbourg, Strasbourg, 67000, France.*

*^4^* *Istituto di Chimica della Materia Condensata e Tecnologie per l'Energia (ICMATE), Consiglio Nazionale delle Ricerche (CNR), 35131 Padova PD, Italy.*

*^5^ Dipartimento di Scienze Chimiche, Università di Padova, 35131 Padova PD, Italy.*

*^6^ Institut de Génomique Fonctionnelle, CNRS UMR 5203, Inserm U1191, Université de Montpellier, 34094 Montpellier, France.*

*^7^ Optical Sciences Division, Code 5600 and ^8^ Center for Bio/Molecular Science and Engineering, Code 6900, U.S. Naval Research Laboratory, Washington, D.C. 20375, United States.*

*^9^* McMaster University, Department of Engineering Physics, Hamilton, ON L8S 4L7, Canada.

* Corresponding author: [tjs@chem.ku.dk](mailto:tjs@chem.ku.dk), [hildebrandt@mcmaster.ca](mailto:hildebrandt@mcmaster.ca)

**PL lifetime fitting.** Multiexponential fits (curves in **Figure 3** in the main manuscript) were performed using the explanations and equations discussed below.

**Tb detection channel:**

$$I\left( t \right)=A_{D}\exp\left( \frac{-t}{\tau_{D}} \right)+A_{DA1}\exp\left( \frac{-t}{\tau_{DA1}} \right)+A_{DA2}\exp\left( \frac{-t}{\tau_{DA2}} \right)+C_{1}$$

Here, the *A* terms are the preexponential factors and *C*_1_ is a constant accounting for background signal offset. The Tb donor PL lifetime in the absence of the acceptor (τ_D_) was first fit using Tb only and a single-exponential fit. This value was the used as a fixed parameter for fitting the FRET-probe PL decay curves. It is necessary to account for the unquenched Tb emission in those curves because distance calculations should only take into account actual FRET pairs, i.e., only FRET-quenched components. These FRET components were fit as another one or two exponentials (τ_DA1_ and τ_DA2_), depending on the quality of fit and with the aim of always using as few exponentials as possible. The average “FRET-lifetime” (<τ_DA_>), i.e., the quenched component, was calculated using amplitude averaging:

$$\left\langle\tau_{DA} \right\rangle=\frac{A_{DA1}\tau_{DA1}{+A}_{DA2}\tau_{DA2}}{A_{DA1}+A_{DA2}}$$

From this average FRET-lifetime, *E*_FRET_ was calculated as follows:

$$E_{FRET}=1-\frac{\left\langle\tau_{DA} \right\rangle}{\tau_{D}}$$

And the distance was calculated as follows:

$$R=R_{0}\left( \frac{\left\langle\tau_{DA} \right\rangle}{\tau_{D}-\left\langle\tau_{DA} \right\rangle} \right)^{1/6}$$

**QD detection channels:**

$$I\left( t \right)=A_{D}\exp\left( \frac{-t}{\tau_{D}} \right)+A_{AD1}\exp\left( \frac{-t}{\tau_{AD1}} \right)+A_{AD2}\exp\left( \frac{-t}{\tau_{AD2}} \right)+A_{AD3}\exp\left( \frac{-t}{\tau_{AD3}} \right)+C_{2}$$

Here, the *A* terms are the preexponential factors and *C*_2_ is a constant accounting for background signal offset. The Tb donor PL lifetime in the absence of the acceptor (τ_D_) was first fit using Tb only and a single-exponential fit. This value was the used as a fixed parameter for fitting the FRET-probe PL decay curves. Although the Tb crosstalk signals in the QD detection channles are much weaker than the Tb signal in the Tb channel, it is necessary to account for the crosstalk of the unquenched Tb emission in those curves because distance calculations should only take into account actual FRET pairs, i.e., only FRET-sensitized components. These FRET components were fit as another three exponentials (τ_AD1_, τ_AD2_, and τ_AD3_) because good qualities of fit could only be accomplished with four exponentials in total. One of the reasons was the relatively strong PL background from direct QD excitation, i.e., the QD PL decay could still be detected on the ms time range because of the very strong QD excitation in the UV. Thus, τ_AD1_ accounted for the QD background, whereas τ_AD2_ and τ_AD3_ were the actual FRET-sensitized components. The average “FRET-lifetime” (<τ_AD_>), i.e., the quenched component, was calculated using amplitude averaging. Owing to the fact that the FRET-lifetimes are FRET-sensitized lifetimes, the single amplitudes must be corrected with *k*_FRET_ before averaging:

$$\left\langle\tau_{AD} \right\rangle=\alpha_{AD2}\tau_{AD2}{+\alpha}_{AD3}\tau_{AD3}$$

with

$$k_{FRETi}={\tau_{ADi}}^{-1}+{\tau_{D}}^{-1}$$

and

$\alpha_{AD2}=\frac{\left( A_{AD2}{/k}_{FRET2} \right)}{\left( A_{AD2}{/k}_{FRET2} \right)+\left( A_{AD3}{/k}_{FRET3} \right)}$	or	$\alpha_{AD3}=\frac{\left( A_{AD3}{/k}_{FRET3} \right)}{\left( A_{AD2}{/k}_{FRET2} \right)+\left( A_{AD3}{/k}_{FRET3} \right)}$

From the average FRET-lifetime, *E*_FRET_ was calculated as follows:

$$E_{FRET}=1-\frac{\left\langle\tau_{AD} \right\rangle}{\tau_{D}}$$

And the distance was calculated as follows:

$$R=R_{0}\left( \frac{\left\langle\tau_{AD} \right\rangle}{\tau_{D}-\left\langle\tau_{AD} \right\rangle} \right)^{1/6}$$

All values determined in both the Tb donor channel and the QD acceptor channels are shown in **Tables S1** and **S2**.

**Table S1.** PL lifetime fitting results of FRET-probe decay curves in the Tb detection channel.

**Table S2.** PL lifetime fitting results of FRET-probe decay curves in the QD detection channels.

**Figure S1:** Representative PL decay curves used for integrating the target-concentration-dependent PL intensities in a time-window from 0.1 to 8.0 ms in order to establish the assay calibration curves shown in **Figure 4** in the main manuscript. Only one dataset is shown per target, whereas the assay calibration curves used triplicate measurements (and 10 repeats for the blank). The different PL decay kinetics for each specific FRET-probe are clearly visible for the different targets.

**Figure S2:** PL decay curves used for integrating the target-concentration-dependent PL intensities in the corresponding time-windows (W1 to W6) in order to establish the assay calibration curves shown in **Figure 5** in the main manuscript.

**Figure S3:** PL decay curves corresponding to 9 different samples (M1 to M9) with mixed DNA target concentrations used for hexaplexed target concentration recovery shown in **Figure 6** in the main manuscript.
